# Autochthonous Neurocysticercosis Brain Lesions Mimicking Metastatic Disease, Spain

**DOI:** 10.3201/eid3207.260587

**Published:** 2026-07

**Authors:** Elena Hernández-Sánchez, Paloma Monllor, Maria Gil-Fortuño, Edelmira Guillamón

**Affiliations:** Hospital de La Plana, Vila-real, Castellón, Spain

**Keywords:** Parasites, neurocysticercosis, *Taenia solium*, autochthonous infection, brain lesions, central nervous system infections, Spain

## Abstract

Autochthonous neurocysticercosis is exceptionally rare in Western Europe. We describe multiple brain lesions, initially mimicking metastases, in a 60-year-old man in Spain without travel history. We confirmed diagnosis by neuroimaging and positive serology. Our study highlights cryptic local *Taenia solium* cestode transmission risks and diagnostic challenges in nonendemic regions.

In 2025, A 60-year-old man, a lifelong resident of Castellón (Valencian Community), Spain, sought treatment for a 2-week history of progressive headache and subtle behavioral changes. He had no history of international travel or immunosuppression. Neurologic examination revealed mild psychomotor slowing without focal deficits. Initial laboratory tests were unremarkable except for an elevated total serum IgE of 200 IU/mL (reference <100 IU/mL). A noncontrast head computed tomography scan revealed multiple ill-defined intra-axial lesions with marked vasogenic edema, initially suspected to represent metastatic disease (Figure, panel A). We initiated dexamethasone (8 mg/d), rapidly resolving his symptoms. Extensive oncologic workup, including whole-body, contrast-enhanced computed tomography, colonoscopy, and fluorine-18 fluorodeoxyglucose positron emission tomography/computed tomography showed no primary malignancy. A subsequent brain magnetic resonance imaging scan demonstrated numerous solid-cystic lesions diffusely distributed throughout both hemispheres, displaying ring enhancement (Figure, panel B). Of note, several cystic lesions contained internal nodular components suggestive of a scolex (Figure, panel C).

**Figure:**
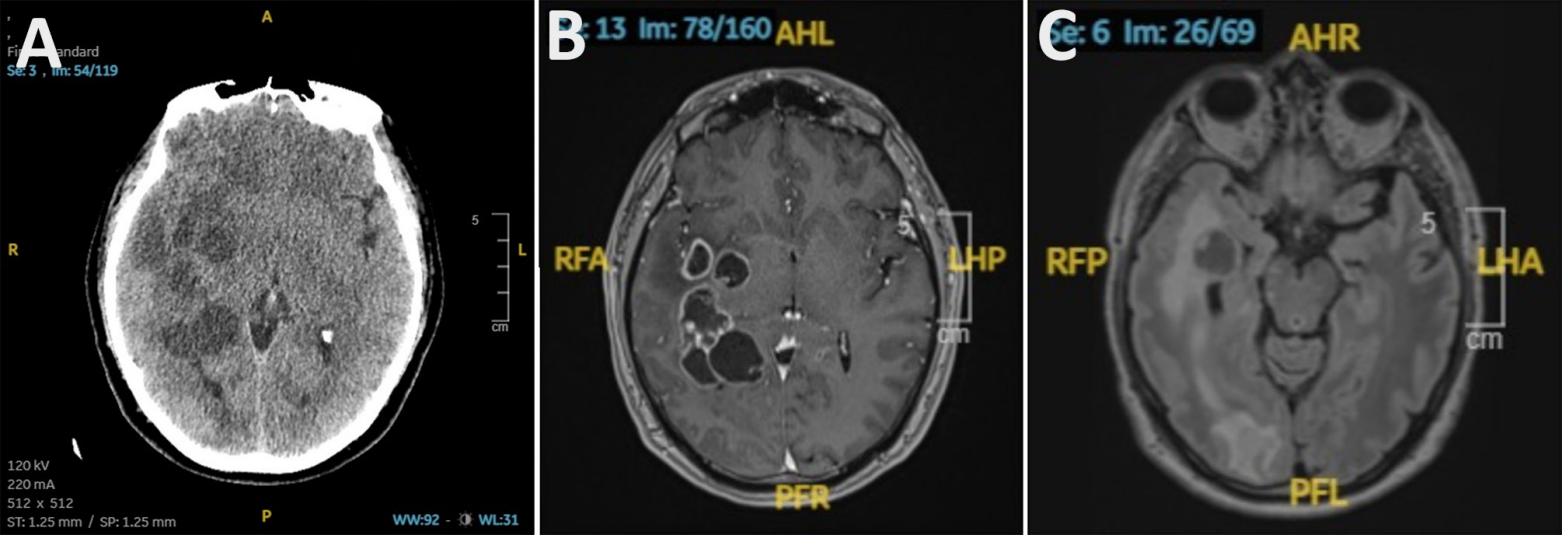
Radiologic findings from a study of autochthonous neurocysticercosis brain lesions mimicking metastatic disease, Spain. A) Noncontrast head computed tomography demonstrating multiple intra-axial lesions with surrounding vasogenic edema. B) Axial T1-weighted magnetic resonance imaging sequence with gadolinium showing multiple ring-enhancing lesions. C) Axial T2-FLAIR magnetic resonance imaging sequence revealing cystic lesions with internal nodular components suggestive of a scolex, surrounded by extensive edema.

Both the patient we report and his household contacts lacked travel history to *Taenia*-endemic regions, and results of stool examinations for ova and parasites for the patient and his household contacts were negative. However, the man had previously worked as a construction laborer until retiring 10 years prior. In that occupation, he frequently shared meals and communal sanitary facilities with migrant coworkers from regions endemic for *Taenia solium* tapeworms, presenting a potential setting for cryptic fecal–oral transmission. 

Given the pathognomonic imaging features and the patient history, we evaluated serum antibodies against *T. solium* by using enzyme-linked immunoelectrotransfer blot at the Spanish National Centre for Microbiology (Instituto de Salud Carlos III, Madrid), the national reference laboratory. The official report confirmed a positive diagnostic result. After noting fulfillment of 2 major Del Brutto diagnostic criteria (*1**,**2*), we established a definitive diagnosis of neurocysticercosis (NCC). We treated the patient successfully with albendazole (400 mg 2×/d) and praziquantel (1,200 mg 3×/d) (*3*), alongside dexamethasone taper, without complications.

In Europe and the United States, NCC is a disease seen predominantly in migrants and returning travelers. Autochthonous transmission is exceptionally rare. In the United States, domestically acquired cases account for <2% of all NCC diagnoses, usually linked to close contact with a household employee or family member from an endemic area (*4*). In Europe, a comprehensive systematic review identified only 18 confirmed autochthonous cases of NCC across Western Europe in 1990–2011 (*5*). More recent data confirm this rarity; during 2000–2019, reports of autochthonous cases across all European Union member states totaled <30 (*6**,**7*). In Spain, Herrador et al. identified 1,912 hospital discharges with cysticercosis during 1997–2014, with hospitalization rates paralleling external migration trends (*8*). 

Our case emphasizes that the absence of travel history should not preclude NCC from the differential diagnosis of multiple ring-enhancing brain lesions, even in regions where metastatic cancer is statistically much more likely. Early recognition of specific neuroimaging markers, such as the scolex, coupled with confirmatory enzyme-linked immunoelectrotransfer blot testing, can prevent unnecessary invasive oncologic procedures and lead to prompt, targeted antiparasitic therapy.
